# Recency and frequency of HIV testing among men who have sex with men in Germany and socio-demographic factors associated with testing behaviour

**DOI:** 10.1186/s12889-015-1945-5

**Published:** 2015-07-30

**Authors:** Ulrich Marcus, Martyna Gassowski, Martin Kruspe, Jochen Drewes

**Affiliations:** Department of Infectious Disease Epidemiology, Robert Koch-Institute, P.O. Box 650261, , 13302 Berlin, Germany; Public Health, Free University, Berlin, Germany

## Abstract

**Background:**

Testing for presence of HIV infection is a pre-requisite to qualify for antiretroviral treatment. A considerable proportion of German men who have sex with men (MSM) infected with HIV have a CD4 cell count below 350 cells/μl at time of diagnosis and are thus defined as “late presenters”. Late presentation increases the risk of adverse disease outcomes. In addition, knowledge and assessment of HIV status is often used for decisions about condom use and anal intercourse with steady and non-steady partners. Incorrect assumptions may result in high risk for HIV transmission.

**Methods:**

Between 11/2013 and 01/2014 MSM were recruited to an online survey predominantly by personalized invitation messages from MSM social networking and dating websites. Respondents were asked about demographic characteristics, HIV testing history, reasons for testing decisions, and sexual behaviours. We describe reasons for not testing and analyse factors associated with not or infrequent testing using univariable and multivariable multinomial regression.

**Results:**

Questions on HIV testing history were answered by 15,297 respondents. An HIV test within the last 12 months was reported by 38 %, a test more than 12 months ago by 27 % and 35 % had never been tested for HIV. Compared to recently tested, respondents who had never tested were more likely to be younger than 25 years (adjusted relative risk ratio (aRRR) 2.90, 95 % CI 2.11-3.99), living in a settlement with less than 100,000 inhabitants (aRRR 1.47, 95 % CI 1.18-1.83), being less open about their sexual orientation to their co-workers/classmates, and particularly to their primary care provider (aRRR 4.54, 95 % CI 4.02-5.11). Untested and less frequently tested respondents reported less sex partners and a lower proportion reported unprotected anal intercourse (UAI) with a non-steady partner (24 % compared to 38 % among those recently tested).

**Conclusions:**

MSM who were younger, who did not live in large cities, and who were not out about their sexual orientation tested less frequently for HIV. Apart from strengthening protection from sexual orientation-related discrimination and empowering MSM who conceal their orientation, more opportunities to test anonymously and without revealing one’s sexual orientation should be provided.

**Electronic supplementary material:**

The online version of this article (doi:10.1186/s12889-015-1945-5) contains supplementary material, which is available to authorized users.

## Background

Early diagnosis of HIV and timely initiation of antiretroviral treatment (ART) are essential to prevent severe clinical consequences of HIV infection. By reducing infectiousness of people receiving ART, early treatment also contributes to reduction of HIV transmission [1]. However, despite availability of HIV testing in regular healthcare institutions and establishment of dedicated anonymous HIV testing sites, modelling of the HIV epidemic among MSM in Germany suggests no tangible reduction of the number of undiagnosed HIV infections in this group (estimated 7,000 – 10,000 undiagnosed MSM, i.e. 15-17 % of all estimated prevalent cases) [2]. A considerable proportion of MSM infected with HIV in Germany has a CD4 cell count below 350 cells/μl at time of diagnosis (45 % as of 2010), indicating a long period between acquisition and diagnosis of the infection [3]. In addition, MSM often use knowledge and assessment of HIV status for decisions regarding condom use and anal intercourse with steady and non-steady partners [4]. Inaccurate knowledge of HIV status may result in increased risk for HIV transmission for either partner.

Most voluntary HIV testing in Germany is conducted in private medical practices. Health insurance covers HIV testing in cases of suspected HIV infection; however, reasons justifying suspicion are not clearly defined and can range from clinical symptom to reported risk behaviour, which could merely be unprotected sexual intercourse. Hence, a restrictive definition by the physician may result in the patient having to pay testing fees out of his own pocket. Another possibility to test for HIV is in public health offices, which are present in all larger cities and offer anonymous and - in general - free HIV testing. However, opening hours of these offices are often inconvenient for people with full-time employment. In recent years, community-based voluntary testing and counselling sites specifically targeting MSM have been established in larger German cities [5]. Due to limited public funding most of them charge testing fees. Blood donation is officially strongly discouraged for MSM [6] but many MSM do so nevertheless by not answering the donor selection questions truthfully and at least a part of them seems to do so primarily to get an HIV test.

Home test kits for self-diagnosis of HIV are not legally approved for marketing in Germany [7], but may be ordered and imported illegally on the internet. Rapid tests are only available for health care institutions. Hospitals play no major role for voluntary testing. In hospitals, HIV tests can be prescribed for differential diagnosis of suspect clinical conditions, or tests may be conducted in cases of accidental percutaneous or mucous membrane exposure to body fluids in order to decide whether HIV- Post-exposure prophylaxis (PEP) should be initiated. Tests may also be conducted for hospitalized pregnant women or after delivery if an HIV test result during pregnancy is not available. Infrequently, tests may be ordered routinely before surgery [8]. Almost all HIV tests conducted in laboratories are based on 4^th^ generation ELISA tests. Blood donations are additionally tested routinely with nucleic acid amplification assays. Dedicated HIV testing sites partly offer 3^rd^ or 4^th^ generation rapid tests, partly laboratory-based tests. In other health care settings rapid tests are only used exceptionally [9].

The most recent and most representative data on HIV testing among German MSM are from the European MSM Internet Survey (EMIS) conducted in 2010. The proportion of men who had received a test result in the last 12 months (recent testing) in Germany in EMIS was 34 % (European Median 34.5 %). In the same study, 30 % of the respondents reported never having tested for HIV (European Median 37 %). Recency of HIV testing was only weakly associated with recency of reported risk for HIV acquisition. In logistic multivariable regression analysis several factors were identified as being independently associated with testing. Ever being tested for HIV was positively associated with age, number of sexual partners in the last 12 months, perceived access to free or affordable HIV testing, and settlement size. Survey respondents being less out about their sexual preferences and having a higher score for internalised homonegativity were less likely to have been tested for HIV. Men who reported living with a steady partner were less likely to have ever been tested, but more likely to have had a recent test [10].

In this analysis we aim to describe the differences in demographic and socio-behavioural characteristics of MSM who have tested for HIV recently or less frequently and MSM who have so far never tested for HIV based on data collected in a new online survey conducted in 2013 (SMA 2013). In addition to items already queried in EMIS 2010, SMA 2013 included a few more variables potentially associated with testing behaviour and testing decisions, and most importantly SMA 2013 included explicit questions on reasons for testing and reasons for not testing. A better understanding of the factors involved in testing decisions may help to develop new approaches to achieve a broader and more up-to-date knowledge of HIV serostatus among MSM.

## Methods

### Study procedures

Data were collected between November 2013 and January 2014 through a nationwide, anonymous online-survey targeting MSM (the SMA 2013 survey). Participants were recruited for the survey through private messages and banners on several social networking and dating sites for gay men. By clicking on a link or banner the participant was referred to the survey’s entry site, which contained information about the goals and contents of the survey, terms of participation and data privacy. By clicking on a button “I have read and understood the information above” the participant gave his informed consent and was referred to the online questionnaire. At the end of the online questionnaire participants were offered a free test voucher for download. More details including a description of the CHERRIES criteria for the survey are provided in the Additional file 1.

The online survey protocol was evaluated and approved by the ethical review board of the Charité University Clinic in Berlin (EA1/266/13). Suggestions by the data protection office of the federal state of Berlin to improve data protection for survey participants were implemented. Participants had to be at least 16 years old. Although less than 18 years old are considered minors in Germany, the ethical review board accepted inclusion of this age group in the study without any additional specific requirements.

### Measures

#### Testing sites, recency and frequency of testing, and reasons for testing and not testing for HIV

Data on HIV testing history (site of last HIV test, recency of last HIV test and frequency of HIV testing) as well as reasons for testing decisions were collected using closed ended questions. Private practices as testing sites were differentiated into practices of doctors known to be gay or with a large proportion of gay clients and other private practices. In addition to the response options provided about reasons for testing, the questions also contained an open ended option (“What other reason did you have?”). Answers to this open ended question were content analysed and either re-coded into existing response options or coded into new response categories. Reasons for not testing were assessed by two different questions: one direct question was asked to participants who reported never testing for HIV or not having been tested in the previous five years. Another question on reasons for refusal was asked to participants who declined the offer of a free test voucher.

#### Reported sexual behaviours and perceived risk of HIV infection

Sexual behaviour was assessed by questions on number, gender and type of partners (steady, non-steady); relationship status (single; monogamous relationship; open relationship); condom use for anal sex; risk management approaches (HIV serostatus communication with steady and non-steady partners); meeting places for partners; and the perceived risk of these behaviours. Perceived risk was assessed by an eleven-point scale from 0 to 10. For the analysis, the eleven point scale was reduced to four risk levels: no risk (0–1), low risk (2–4), moderate risk (5–7), and high risk (8–10).

### Psychological scales

#### Internalized homonegativity

Internalized homonegativity is defined as the extent to which gay men agree with negative societal attitudes about homosexuality. To assess internalized homonegativity we used the established scale by Smolenski et al. [11]. This scale contains 8 items (e.g. “Even if I could change my sexual orientation, I wouldn’t”) with a seven-point Likert-type scale (totally agree – totally disagree). Reliability of this scale was good, with a Cronbach’s α of .78. For the analysis we condensed the seven point score to three levels, representing low, middle and high levels of internalized homonegativity.

#### HIV-related stigma

Stigmatizing attitudes were assessed using a self-constructed ad-hoc scale with six items (e.g. “HIV-positive people are irresponsible”, “I wouldn’t want to be in a relationship with an HIV-positive individual”). Responses used a four-point Likert-type scale (totally agree – totally disagree). The scale’s reliability was good, with a Cronbach’s α of .74. For the analysis we condensed the six point scale to three levels, representing low, middle, or high stigmatizing attitudes towards HIV.

#### Anticipated HIV stigma

Anticipated HIV stigma can be understood as expectations about the experience of stigma in case of being tested HIV positive. Anticipated HIV stigma was assessed with six self-constructed items (e.g. “My family would be disappointed with me”, “I would get trouble in my job”. Responses were recorded on a four-point Likert-type scale (very likely – very unlikely). Reliability of this scale was very good with a Cronbach’s α of .84. For the analysis we condensed the six point scale to three levels of low, middle, and high stigma anticipation in case of an HIV diagnosis.

More details on the items used in the psychological scales are provided in the Additional file 1.

### Demographic and other variables

Demographic characteristics used in the analysis were age, education level (high school or less), type of work (blue collar worker; white collar worker/public official/self-employed; student/trainee), monthly equivalence income (<936€; 936–1,895€; >1,895€) [12], and settlement size (<100,000 inhabitants; 100,000-1,000,000; >1,000,000). Ethnicity or nationality of respondents was not queried.

Other variables used in the analysis were outness towards co-workers/classmates and towards primary health care provider about sexual orientation (less than half know; half or more know; not applicable); gay subculture involvement (frequently visiting social venues; frequently visiting sex venues; frequently visiting both social and sex venues; infrequently/never visiting gay venues – a more detailed description of this variable is given in the Additional file); and having been reached by the national HIV prevention campaign for MSM (IWWIT).

### Statistical analysis

The analysed subsample included all men who had answered the question about ever testing for HIV and who had never received a positive HIV test result. For the purpose of this analysis three different HIV testing statuses were defined: recently tested, comprising men who reported testing for HIV within the previous 12 months; distantly tested, comprising men who reported testing for HIV more than 12 months ago; never tested, comprising men who reported never to have tested for HIV.

Associations of testing status with the variables described above were analysed using univariable and multivariable multinomial logistic regression. HIV-testing status was used as the outcome variable, with “Recently tested” set as reference. In a first step, we performed a univariable analysis with the variables age, educational level, occupational status, equivalent-income, settlement size, sexual attraction, outness towards co-workers/classmates, outness towards primary health care provider, gay subculture involvement, internalised homonegativity, HIV-related stigma, anticipated HIV stigma, familiarity with the IWWIT-campaign, relationship status, number of non-steady UAI-partners, and assessment of the own risk. Using a stepwise forward selection approach, where a p-value of <0.05 was considered to indicate statistical significance, a multivariable model was constructed. We also tested for interaction between the variables age and settlement size and the variables outness towards co-workers/classmates, readiness to HIV stigmatization, gay subculture involvement, and relationship status. Interaction terms found to improve the model significantly, using the likelihood-ratio test, were included in the final model.

All analyses were performed using the statistical software StataSE12.

## Results

The survey sample consisted of 16,734 men. Among them 1,437 reported a previous positive HIV test result and were excluded from this analysis, leaving an analytic sample of 15,297 participants.

### Recency, frequency, and reasons for testing and not testing for HIV

An HIV test within the last 12 months was reported by 38 % of the respondents, a test more than 12 months ago by 27 % and 35 % had never been tested for HIV.

The free testing voucher offered at the end of the survey was accepted by 3,603 participants, representing 29 % of all participants finalizing the questionnaire.

**Fig. 1:**
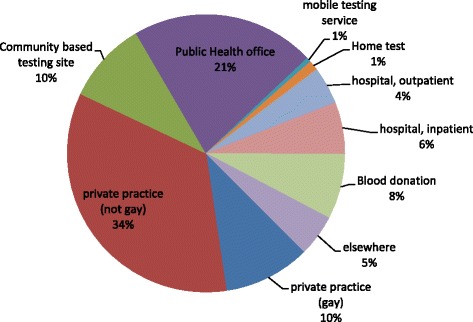
Place of last HIV test, German MSM online survey 2013

The distribution by last testing site is presented in Fig. 1. The most frequently reported testing sites were private practices (44 %, both gay and non-gay practitioners) and designated HIV testing services (32 %, public health offices, community based testing sites, mobile testing services). Home test use war reported by merely 1 %.

Recency of the last HIV test is presented in Table 1. Of those who had ever tested, 60 % had been tested within the previous 12 months. Reasons for the last HIV test and frequency of previous testing are presented in Table 2, stratified by HIV testing status. The number of previous tests was higher among recent testers compared with distant testers, with 33 % of the recent and 7 % of the distant testers having tested for HIV at least five times. Routine testing was the most common reason for testing among recent testers (53 %), followed by testing after a perceived transmission risk (42 %). Among distant testers testing after a perceived transmission risk was the most common reason (40 %), followed by ‘relationship’ testing (29 %).

**Table 1:** Recency of last HIV test among German MSM (not having been diagnosed with HIV) responding to an online survey in 2013

	N	% of ever tested	% of total
Recent 6 months	3462	36.0	22.5
Recent 12 months	2333	23.6	15.3
1-5 years ago	2701	27.3	17.7
More than 5 years ago	1300	13.1	8.5
Ever tested	9886	100.0	64.9
Never tested	5340		35.1
Total	15226		100.0

**Table 2:** Reasons for last HIV test, frequency of testing, and distance to last transmission risk event, German MSM online survey 2013

Reasons for last HIV test (multiple answers possible)	Proportion of respondents indicating the specific reason	
	Recent (<=12 months) (*N* = 5,885)	Distant (>12 months) (*N* = 4,001)
Transmission risk event within 6 months preceding the last test	29.4 %	26.5 %
Transmission risk event longer than 6 months ago	12.7 %	13.1 %
Symptoms suggestive of acute HIV infection	3.1 %	1.9 %
Symptoms suggestive of compromised immune status (AIDS)	2.3 %	2.4 %
Forced to get tested	1.5 %	2.9 %
Recommended to get tested	10.8 %	11.6 %
To be able to have sex without condom with partner	21.1 %	29.9 %
Routine testing	53.4 %	13.1 %
Other reasons	24.0 %	32.9 %
Lifetime frequency of having tested for HIV	(*N* = 5,861)	(*N* = 3,992)
Once	14.3 %	41.5 %
Twice	14.9 %	24.0 %
3 times	15.6 %	15.9 %
4 times	22.2 %	12.0 %
5 or more times	32.9 %	6.6 %
Interval between last transmission risk event and last HIV test^a^		
	Recent (N = 1,728)	Distant (N = 1,061)
<=4 weeks	18.2 %	11.8 %
4 weeks – 12 weeks	26.3 %	25.1 %
>12 weeks	47.5 %	46.8 %
Don’t remember	8.1 %	16.3 %
Total	100.0 %	100.0 %

^a^Only men who reported a transmission risk within 6 months preceding the test

If the last HIV test had been conducted due to a preceding potential risk of infection and within six months after the transmission risk event, the time interval to the respective event was queried (Table 2). Of those who answered this question, between 12 % and 18 % had tested within 4 weeks, resembling between 3 % and 5 % of all distant or recent testers. Particularly for those who have been tested with rapid tests, 4 weeks may not be sufficient time to reliably detect an HIV seroconversion.

### Reasons for not or infrequent testing

Reasons for never testing or for not having been tested in the previous five years are presented in Table 3, stratified by testing status. The most common reason for not testing was the lack of perceived risk (71 % of those never tested, 65 % of those not tested in the previous 5 years). The lack of perceived risk was also the most common reason to decline an offer of a free testing voucher offered at the end of the survey (55 % resp. 50 %). Not wanting to know one’s HIV status was mentioned infrequently as a reason for not testing (5 % of those never tested and 3 % of those distantly tested).

**Table 3:** Reasons for not having tested recently or never and reasons for not accepting test vouchers, German MSM online survey 2013

Reasons for not having tested in the recent 5 years or for never having tested for HIV (multiple answers possible)	Proportion of respondents indicating the specific reason	
	No test in the previous 5 years (*N* = 1,442)	Never tested (*N* = 5,271)
Didn’t take any risks (since previous test)	64.6 %	71.4 %
Despite taking some risks I don’t believe to have been infected	17.2 %	21.6 %
Believe to be HIV-negative because my partner has tested negative	8.2 %	6.6 %
Afraid of getting a positive test result	6.5 %	12.5 %
Don’t like to talk about the sex I have	2.6 %	8.6 %
Don’t want to be judged for the sex I have	3.4 %	7.3 %
Waiting time	3.9 %	8.2 %
Concerned about anonymity/confidentiality	3.4 %	9.2 %
No nearby adequate testing site	2.1 %	3.7 %
test too expensive	1.8 %	4.9 %
Don’t want to know my status	2.9 %	5.1 %
no need to rush	0.7 %	2.8 %
Other reasons	14.8 %	18.4 %
Reasons for not accepting free testing voucher	Previous test (*N* = 2048)	Never tested (*N* = 2368)
Don’t believe to be at risk	50.3 %	54.8 %
Too burdensome to get to the testing site	29.9 %	35.1 %
No health complaints	19.3 %	27.1 %
Currently don’t want to know	5.0 %	10.1 %

Other factors mentioned by distant and never testers as barriers for (more frequent) testing were geographic distance from next free HIV testing site; HIV testing fees requested from primary care physicians; perceived lack of anonymity in designated HIV testing sites; perceived lack of confidentiality regarding sexual risks and sexual orientation when testing in regular health care settings.

### Associations between testing status and other variables

The distribution of respondents by a number of socio-demographic and behavioural characteristics, stratified by HIV testing status, is presented in Table 4. Table 5 presents univariable and adjusted multivariable relative risk ratios (RRR, adjusted for interaction terms age x settlement size; age x relationship status; age x subculture involvement) for these variables, comparing those distantly and never tested with those recently tested for HIV.

**Table 4:** Distribution of respondents by socio-demographic and behavioural characteristics, stratified by HIV testing status, German MSM online survey 2013

		Last HIV test within the past 12 months (n=5,892)	Last HIV test more than 12 months ago (n=4,001)	Never tested for HIV (n=5,341)
		n (%)	n (%)	n (%)
Age (years)	*16-24*	926 (15.7)	271 (6.8)	1,979 (37.1)
	*25-34*	1,574 (26.8)	1,032 (25.8)	1,291 (24.2)
	*35-44*	1,455 (24.7)	1,158 (29.0)	869 (16.3)
	*45-54*	1,380 (23.5)	1,066 (26.7)	777 (14.6)
	*>54*	550 (9.4)	473 (11.8)	423 (7.9)
Educational level	*Low*	2,278 (38.9)	1,427 (35.9)	2,071 (39.0)
	*High*	3,578 (61.1)	2,554 (64.2)	3,239 (61.0)
Occupational status	*Blue-collar worker*	318 (7.3)	208 (6.9)	357 (9.8)
	*White-collar /self-employed*	3,294 (76.1)	2,476 (81.9)	2,068 (56.6)
	*Other (incl. student/trainee)*	719 (16.6)	341 (11.3)	1,230 (33.7)
Equivalent-income	*Less than 936€*	791 (20.6)	436 (15.8)	959 (28.9)
	*936€ to 1 895.99€*	1,352 (35.3)	931 (33.7)	1,306 (39.4)
	*1 896€ or more*	1,689 (44.1)	1,396 (50.5)	1,054 (31.8)
Settlement size	*<100 000 inhabitants*	2,660 (45.3)	1,800 (45.2)	3,046 (57.4)
	*100 000–1 000 000 inhabitants*	1,885 (32.1)	1,341 (33.6)	1,572 (29.6)
	*>1 000 000 inhabitants*	1,325 (22.6)	846 (21.2)	686 (12.9)
Sexual attraction	*Men mostly/only*	5,173 (87.9)	3,465 (86.6)	4,208 (78.8)
	*Men and women equally*	369 (6.3)	242 (6.1)	520 (9.7)
	*Women mostly/only*	346 (5.9)	296 (7.4)	612 (11.5)
Outness towards co-workers/classmates	*More than half know*	3,083 (54.0)	2,015 (51.0)	1,610 (30.6)
	*Less than half know*	1,382 (23.8)	929 (23.5)	1,310 (24.9)
	*Nobody knows/nonexistent*	1,348 (23.2)	1,004 (25.4)	2,336 (44.4)
Outness towards primary health care provider	*Yes*	3,335 (60.9)	1,893 (50.8)	898 (18.9)
	*No*	2,146 (39.2)	1,834 (49.2)	3,864 (81.1)
Gay subculture involvement	*Frequently visiting social venues*	1,720 (29.2)	1,028 (25.7)	1,213 (22.8)
	*Freq. visit.both social and sex ven.*	1,393 (23.7)	544 (13.6)	411 (7.7)
	*Frequently visiting sex venues*	808 (13.7)	578 (14.5)	652 (12.2)
	*Infreq./never visiting gay venues*	1,961 (33.3)	1,847 (46.2)	3,051 (57.3)
Internalised homonegativity	*Low*	4,420 (79.3)	2,916 (77.4)	3,142 (65.0)
	*Middle*	888 (15.9)	655 (17.4)	1,353 (28.0)
	*High*	263 (4.7)	195 (5.2)	338 (7.0)
HIV-related stigma	*Low*	3,875 (73.7)	2,521 (70.2)	2,644 (58.2)
	*Middle*	1,243 (23.6)	972 (27.1)	1,640 (36.1)
	*High*	141 (2.7)	99 (2.8)	256 (5.6)
Anticipated HIV stigma	*Low*	1,561 (30.0)	988 (28.1)	1,062 (23.7)
	*Middle*	2,639 (50.7)	1,837 (52.2)	2,288 (51.1)
	*High*	1,006 (19.3)	692 (19.7)	1,126 (25.2)
Familiarity with the IWWIT-campaign	*Yes*	2,994 (68.1)	1,866 (60.9)	1,578 (42.4)
	*No*	1,402 (31.9)	1,199 (39.1)	2,145 (57.6)
Relationship status	*Single*	2,975 (50.6)	1,583 (39.6)	3,147 (59.0)
	*In a monogamous relationship*	502 (8.5)	720 (18.0)	589 (11.0)
	*In an open relationship*	2,398 (40.8)	1,695 (42.4)	1,598 (30.0)
Number of non-steady UAI-Partners	*0 men*	3,258 (61.8)	2,792 (77.3)	3,487 (75.6)
	*1-10 men*	1,885 (35.8)	778 (21.6)	1,079 (23.4)
	*11 men*	129 (2.45)	40 (1.1)	46 (1.0)
Assessment of the own risk	*None*	1,175 (23.9)	1,319 (38.9)	1,848 (43.0)
	*Low*	2,985 (60.7)	1,775 (52.3)	2,072 (48.2)
	*Medium*	604 (12.3)	251 (7.4)	307 (7.2)
	*High*	150 (3.1)	47 (1.4)	68 (1.6)

**Table 5:** Univariable and adjusted^a^ multivariable multinomial regression analysis results for recently vs. distantly and recently vs. never tested for HIV; German MSM online survey 2013

		Recently tested for HIV vs. distantly tested for HIV		Recently tested for HIV vs. never tested for HIV		Recently tested for HIV vs. distantly tested for HIV		Recently tested for HIV vs. never tested for HIV	
		Crude RRR	95 % CI	Crude RRR	95 % CI	Adjusted RRR^a^	95 % CI	Adjusted RRR^a^	95 % CI
Age (years)	*16-24*	0.45	0.38-0.52	2.61	2.34-2.90	0.57	0.38-0.86	2.90	2.11-3.99
	*25-34*	1.00		1.00		1.00		1.00	
	*35-44*	1.21	1.09-1.36	0.73	0.65-0.81	1.82	1.31-2.51	0.94	0.64-1.36
	*45-54*	1.18	1.05-1.32	0.69	0.61-0.77	1.93	1.35-2.78	0.90	0.58-1.40
	*>54*	1.31	1.13-1.52	0.94	0.81-1.09	1.63	0.97-2.73	0.96	0.51-1.80
Educational level	*Low*	0.88	0.81-0.95	1.00	0.93-1.08				
	*High*	1.00		1.00					
Occupational status	*Blue-collar worker*	0.87	0.73-1.04	1.79	1.52-2.10				
	*White-collar worker/public servant/self-employed*	1.00		1.00					
	*Other (incl. student/trainee)*	0.63	0.55-0.73	2.72	2.45-3.03				
Equivalent-income	*Less than 936€*	0.80	0.69-0.92	1.26	1.11-1.42				
	*936€ to 1 895.99€*	1.00		1.00					
	*1 896€ or more*	1.20	1.08-1.34	0.65	0.58-0.72				
Settlement size	*<100 000 inhabitants*	0.95	0.87-1.04	1.37	1.26-1.49	1.07	0.86-1.33	1.47	1.18-1.83
	*100 000–1 000 000 inhabitants*	1.00		1.00		1.00		1.00	
	*>1 000 000 inhabitants*	0.90	0.80-1.00	0.62	0.55-0.70	1.14	0.89-1.46	0.89	0.68-1.18
Sexual attraction	*Men mostly/only*	1.00		1.00					
	*Men and women equally*	0.98	0.83-1.16	1.73	1.51-1.99				
	*Women mostly/only*	1.28	1.09-1.50	2.17	1.89-2.50				
Outness towards co-workers/classmates	*More than half know*	1.00		1.00		1.00		1.00	
	*Less than half know*	1.03	0.93-1.14	1.82	1.65-2.00	0.90	0.80-1.02	1.10	0.96-1.25
	*Nobody knows/nonexistent*	1.14	1.03-1.26	3.32	3.03-3.63	0.77	0.66-0.89	1.46	1.27-1.69
Outness towards primary health care provider	*Yes*	1.00		1.00		1.00		1.00	
	*No*	1.51	1.38-1.64	6.69	6.11-7.32	1.79	1.60-2.00	4.54	4.02-5.11
Gay subculture involvement	*Frequently visiting social venues*	1.00		1.00		1.00		1.00	
	*Frequently visiting both social and sex venues*	0.65	0.58-0.74	0.42	0.37-0.48	0.90	0.55-1.49	1.47	0.77-2.79
	*Frequently visiting sex venues*	1.20	1.05-1.37	1.14	1.01-1.30	1.47	0.89-2.44	1.67	0.89-3.13
	*Infrequently/never visiting gay venues*	1.58	1.43-1.74	2.21	2.01-2.42	1.87	1.19-2.94	2.30	1.29-4.10
Internalised homonegativity	*Low*	1.00		1.00		1.00		1.00	
	*Middle*	1.12	1.00-1.25	2.14	1.95-2.36	1.06	0.92-1.22	1.17	1.02-1.34
	*High*	1.12	0.93-1.36	1.81	1.53-2.14	1.03	0.82-1.30	1.26	1.00-1.58
HIV-related stigma	*Low*	1.00		1.00		1.00		1.00	
	*Middle*	1.20	1.09-1.33	1.93	1.77-2.11	1.06	0.94-1.19	1.24	1.11-1.39
	*High*	1.08	0.83-1.40	2.66	2.15-3.29	1.06	0.77-1.46	1.44	1.08-1.91
Anticipated HIV stigma	*Low*	1.00		1.00					
	*Middle*	1.10	1.00-1.21	1.27	1.16-1.40				
	*High*	1.09	0.96-1.23	1.65	1.47-1.85				
Familiarity with the IWWIT-campaign^b^	*Yes*	0.73	0.66-0.80	0.34	0.31-0.38				
	*No*	1.00		1.00					
Relationship status	*Single*	1.00		1.00		1.00		1.00	
	*In a monogamous relationship*	2.70	2.37-3.07	1.11	0.97-1.26	1.43	0.87-2.35	1.14	0.63-2.60
	*In an open relationship*	1.33	1.22-1.45	0.63	0.58-0.68	1.42	1.03-1.97	1.06	0.74-1.54
Number of non-steady UAI-partners^c^	*0 men*	1.00		1.00		1.00		1.00	
	*1-10 men*	0.48	0.44-0.53	0.54	0.49-0.58	0.65	0.58-0.74	0.64	0.57-0.73
	*11 men*	0.36	0.25-0.52	0.33	0.24-0.47	0.71	0.46-1.09	0.93	0.59-1.46
Assessment of the own risk^c^	*None*	1.00		1.00		1.00		1.00	
	*Low*	0.53	0.48-0.58	0.44	0.40-0.48	0.66	0.59-0.74	0.54	0.48-0.61
	*Medium*	0.37	0.31-0.44	0.32	0.28-0.38	0.60	0.49-0.73	0.46	0.38-0.57
	*High*	0.38	0.20-0.39	0.29	0.21-0.39	0.51	0.35-0.75	0.50	0.34-0.73

^a^Additionally adjusted for the following interaction terms: Age x Settlement size; Age x Relationship status; Age x Gay subculture involvement

^b^Nationwide educational campaign about HIV/STI and safe sex practices specifically targeting MSM, launched by the Deutsche AIDS-Hilfe in 2008

^c^In the past 12 months

In the multivariable model the variables education level, professional status, equivalent-income, sexual attraction, anticipated HIV stigma, and familiarity with the national HIV prevention campaign for MSM (IWWIT) were no longer statistically significant. Age, settlement size, outness towards co-workers/classmates, outness towards primary health care provider, gay subculture involvement, internalized homonegativity, readiness to HIV stigmatization, relationship status, number of non-steady unprotected anal intercourse (UAI)-partners in the last 12 months, and perception of the own risk for HIV infection remained as independently significant factors in the model.

Compared to recent testers, never tested men were younger and lived more often in places with less than 100,000 inhabitants, while distantly tested men were more often older and did not differ in terms of settlement size. Never tested men more often reported that none of their co-workers or classmates was aware of their sexual orientation and that their primary health care provider was not aware of it, while distantly tested men were more likely to be out to any of their co-workers and classmates, but also in this constellation the primary health care provider was less likely to be aware of the sexual orientation of the respondent. Among never tested men the internalised homonegativity score, and the readiness to stigmatize people with HIV, were higher than in the group with recent testing, while distant testers were in this regard not different from recent testers. Distant testers were currently more often living in an open relationship. Distant and never testers both reported less non-steady UAI partners und self-assessed their risk for HIV lower than men who had recently been tested.

## Discussion

The most common self-reported reason for never or distant testing was a lack of perceived risk. Lower risk perception in distant and never testers was indeed associated with a lower proportion of respondents reporting non-steady UAI partners in the previous 12 months compared to recent testers. However, 23 % of distant testers and 24 % of never testers still reported UAI in the previous 12 months, compared to 38 % of recent testers. Thus, a lower number of UAI partners can only partly explain why a large proportion of MSM have never tested or tested infrequently.

Distant testers were slightly older than recent testers, but – controlled for other factors like settlement size – did not differ from recent testers in terms of education level, occupation, internalised homonegativity, readiness to stigmatize people with HIV, anticipated stigma, and knowledge of the national HIV prevention campaign for MSM. They reported less non-steady UAI partners, self-assessed their risk for HIV as lower, were less involved in the gay subculture, were less out towards their primary care provider, and more often lived in an open relationship compared to recent testers.

Thus, lower testing frequency may at least partly be explained by lower partner numbers and higher proportions currently living in a steady relationship compared to recent testers. Still, even considering the lower number of UAI partners, assessment of the own risk was lower than for recent testers. This may reflect a perceived better knowledge of the partners, who were less often met in the context of gay venues. It may also be due to other factors which have an impact on individual risk assessments. More, also qualitative research should look into the determinants of HIV risk perception with sexual partners.

Notably, rather than high perceived risk, the most common reason given for recent testing was routine testing.

Factors associated with never testing in EMIS and other published studies, such as lower age, smaller settlement size, not being out towards family/(heterosexual) friends/co-workers, and a higher internalised homonegativity score, were confirmed also in this survey [13–15]. In addition, compared to recent testers never testers rarely visited gay venues and they also had a higher level of stigmatising attitudes towards people with HIV. Similar to distant testers never testers reported lower partner numbers, but this did not fully explain their lower self-assessment of risk.

Substantial proportions of never or distant testers selected reasons such as fear of getting a positive test result, concerns about the anonymity and confidentiality of the testing procedure and result, feeling uncomfortable discussing their sexual behaviours and risks with a counsellor or health care provider, and accessibility and cost issues from the provided list of possible reasons. Perceived barriers or inconvenience of accessing testing sites were also frequently mentioned as reasons for not accepting a free testing voucher. Only a small proportion explicitly indicated not being interested in their HIV status. This is suggestive of a sub-population of not openly gay, often younger men, more frequently living outside of larger cities, and less well connected to the gay subculture. This subpopulation may not be reached well by MSM testing sites in larger cities, and they may be afraid of testing in local public health offices or at their primary care provider because of confidentiality concerns.

About 40 % of the survey respondents with a testing history have used anonymous or known gay-friendly testing sites, which are concentrated in larger cities. Lower testing rates in rural areas and using blood donations as a means to get tested for HIV are likely associated with a relative lack of anonymous or perceived confidential, and gay-friendly testing opportunities outside of the largest cities.

About 50 % of men who reported UAI in the last 12 months with a non-steady partner also reported testing in the last 12 months (see Table 4, Number of non-steady UAI-partners). Other studies have shown, however, that up to 45 % of men who had an as yet undiagnosed HIV infection reported a negative test within the previous 12 months [16]. Thus, even a 50 % rate of recent testing may not provide adequate safety for men who believe that mutual disclosure of their last HIV negative test results with a non-steady partner reduces their risk of HIV infection through UAI.

Some limitations of this analysis should be kept in mind: although online samples of MSM often cover a relatively broad section of the MSM population, they are not representative. For example, MSM with lower socio-economic status are usually underrepresented in MSM surveys. Participation biases may have been accentuated by a relatively high attrition in this survey – close to fifty percent of the respondents who answered the first questions did not finish the questionnaire. No assessment for duplicate or fraudulent data was made. However, due to the relatively long time required to fill in the questionnaire, in combination with the technical problems with the website on which the survey was hosted (see Additional file for more details), and the lack of any material incentives, we believe that duplicate or fraudulent data entry is not very likely and would have no impact on the survey findings. Since all data were self-reported, the usual limitations of self-reported data such as recollection bias and social desirability bias need to be recognized.

## Conclusions

We observed differences in testing frequency of MSM participating in the online survey among men with different socio-demographic and behavioural characteristics. There is evidence that higher sexual risks are associated with more frequent HIV testing. However, despite frequent testing many recently tested men may still not test frequently enough to prevent onward transmission of HIV in case they become infected due to high partner turnover. While testing rates and testing frequencies of MSM in Germany are significantly higher than in the general population (11 % testing in the last 12 months, including blood donor testing [17]), the current level of testing rates and testing frequency among MSM are still too low to prevent a high proportion of late diagnoses and late presentations for care, and to prevent frequent onward transmission of HIV among MSM when they practice condomless sex with HIV serosorting, considering the reported levels of partner numbers and unprotected anal intercourse [18].

Based on our analysis we would recommend to further increase the capacity and promote the existing designated HIV testing sites. We identified accessibility and cost barriers for testing in the regular health care system. Removing these barriers by guaranteeing coverage of one HIV test per year by statutory health insurance independent of declared risk should be considered. This would recognize that some people perceive outing as MSM towards their primary care provider as a barrier for testing, but it would require a revision of current reimbursement regulations. If risks are declared, of course more tests must be covered, as is currently the case already. Testing needs to be made more convenient if we want to increase testing frequency. One in Germany so far unused approach is making home collection or home testing accessible. While marketing of home tests is currently not allowed in Germany, home collection testing would be feasible in the existing legal framework. Home collection testing with the option of receiving the test result by phone would also increase testing options particularly for young and rural MSM. This could easily be promoted online and would thus also better reach MSM who are less involved in the gay subculture. This approach has been tested in the United Kingdom, demonstrating considerable demand among MSM for such a testing option [19]. Also, experiences in other European countries with liberalization of access to HIV home tests should be closely monitored to evaluate whether concerns voiced in Germany regarding referral to counselling and into care are justified. Pilot studies in Germany with targeted distribution of HIV home tests for specific subgroups should be considered to evaluate whether this approach is acceptable, reaches the target population, and increases testing frequency among subgroups at very high risk for infection. Broader implementation of home tests would require revoking the current ban on unrestricted marketing of HIV home tests.

The important role of concerns regarding revealing sexual orientation as barrier for HIV testing argues for the continuation and intensification of efforts to reduce social stigma attached to sexual minorities and to empower MSM who feel the need to conceal their sexual orientation. Finally, high prevalence of stigmatizing attitudes towards people living with HIV seem to play a role as barriers for HIV testing, which is an additional reason to address HIV-related stigma in campaigns targeting not only the general population, but also the gay community.

## Additional file

Additional file 1:
**Description of the online survey using the Checklist for Reporting Results of Internet E-Surveys (CHERRIES*).** File also includes more detailed descriptions of scales and other measures used in the analysis. *[Eysenbach G. Improving the Quality of Web Surveys: The Checklist for Reporting Results of Internet E-Surveys (CHERRIES). J Med Internet Res. 2004 Jul-Sep; 6(3): e34. Published online 2004 Sep 29. doi:10.2196/jmir.6.3.e34].
