# Supplementary material for: Recency and frequency of HIV testing among men who have sex with men in Germany and socio-demographic factors associated with testing behaviour
Source: BMC Public Health. 2015 Jul 30;15:727. doi: 10.1186/s12889-015-1945-5 (PMC4520089; doi:10.1186/s12889-015-1945-5)
Supplement: Additional file 1: — Description of the online survey using the Checklist for Reporting Results of Internet E-Surveys (CHERRIES*). File also includes more detailed descriptions of scales and other measures used in the analysis. *[Eysenbach G. Improving the Quality of Web Surveys: The Checklist for Reporting Results of Internet E-Surveys (CHERRIES). J Med Internet Res. 2004 Jul-Sep; 6(3): e34. Published online 2004 Sep 29. doi:10.2196/jmir.6.3.e34]. [file 12889_2015_1945_MOESM1_ESM.pdf]

Supplemental file

CHERRIES criteria and additional details for German MSM online survey 2013 (SMA 2013)

Content

CHERRIES criteria for SMA 2013 online survey	*page 1-4*
Annex I: Entry page of the online questionnaire	*page 5-6*
Annex II: Invitation mail for men with a profile on planetromeo.com	*page 7*
Details on Scales and other survey measures	*page 8-9*

CHERRIES criteria for SMA 2013

Design

The survey was designed as a nationwide, anonymous online-survey targeting MSM. Participants were recruited for the survey through private messages and banners on several social networking and dating sites for gay men. Private messages were sent to all site members having a profile in German language and residing in Germany. Thus the resulting sample was a convenience sample.

IRB (Institutional Review Board) approval and informed consent process

The online survey protocol was evaluated and approved by the ethical review board of the Charité University Clinic in Berlin (EA1/266/13).

Informed consent

The survey's entry site contained information about who the investigator was, the goals and contents of the survey, terms of participation, data privacy, and approximate length of time of the survey. By clicking on a button "I have read and understood the information above" the participant gave his informed consent and was referred to the online questionnaire (for the information included on the entry site see Annex I).

Data protection

We did not collect any personal data which would allow the identification of participants. Several suggestions by the data protection office of the federal state of Berlin to improve data protection for survey participants were implemented.

Development and pre-testing

The questionnaire was developed by using items of former German surveys with this population. The questionnaire used questions from the 2010 European MSM Internet survey (www.emis-project.eu) as much as possible. Several new questions and scales were included. Experts and stakeholders of the target group were asked to evaluate the questionnaire. The survey

was informally pretested for technical functionality, usability and wording with members of the target population.

Recruitment process and description of the sample having access to the questionnaire

Advertising the survey

The survey was announced on several homepages (dating sites and news sites) directed at the target population. On most homepages a banner or texts were provided with a link to the questionnaire. One large dating site for MSM (planetromeo.com; number of active profiles in Germany as of March 18, 2015: 433,781. More than one profile per person is possible. Estimated number of MSM aged 15-64 years living in Germany as of 2010: approximately 656,000 [Marcus U, et al. Estimating the size of the MSM populations for 38 European countries by calculating the survey surveillance discrepancies (SSD) between self-reported new HIV diagnoses from the European MSM internet survey (EMIS) and surveillance reported HIV diagnoses among MSM in 2009. BMC Public Health 2013, 13:919 http://www.biomedcentral.com/1471-2458/13/919]) sent out a message to every German member with a link to the questionnaire asking the members to participate in the survey (for the wording of the message see Annex II). The survey was announced on planetromeo.com in a time-staggered manner (eight batches, ~50,000 profiles each), originally with the intention to prevent excessive demand for free testing at the cooperating testing sites, where free test vouchers offered at the end of the questionnaire could be used. Due to the lower than expected demand this turned out to be unnecessary. However, unexpectedly the capacity of the server of the survey website was not sufficient to manage the demand, so that long waiting times for users resulted and on some of the first days the survey was practically dysfunctional.

The survey was not specifically adapted for smartphone users. The main recruitment website offers traditional online websites as well as a gps-based smartphone app to manage user profiles. Both types of clients were invited to participate in the survey, however, the lack of smartphone-adaptation was mentioned in the invitation mail. Due to the lack of smartphone adaptation and the technical server problems it is very likely that the survey was filled in preferentially with a personal computer online instead by smartphone. Compared with the previous online survey (EMIS 2010) younger age groups (25-35 years) were less well represented among respondents, which may be due to the higher frequency of app-use in this age group.

Survey administration

The survey was a Web-based survey which was filled in online. Responses were automatically captured and directly stored in a database.

Context

See above.

Mandatory/voluntary

Participation in the survey was voluntary.

Incentives

No incentives were offered.

Time/Date

Data were collected between November 2013 and January 2014

Randomization of items or questionnaires

Randomization or alternating of items was not used.

Adaptive questioning

Adaptive questioning was used throughout the questionnaire to reduce number and complexity of the questions. E.g. separate questions were asked to respondents who indicated that they had received an HIV diagnosis and those who didn't.

Number of Items and screens (pages)

The questionnaire included 344 items and 218 questions, presented online on approximately 100 pages. Due to the adaptive design of the questionnaire the actual number of pages that were seen by an average respondent was much less.

Completeness check

No consistency or completeness check was implemented before the questionnaire was submitted. Several questions (e.g. gender, age, HIV test, etc.) were regarded as especially important. In case the respondent didn't answer one of these questions, they were reminded using a pop-up window, to answer this question. If the respondent still was not willing to answer the question he was able to continue the questionnaire.

Review step

Respondents were able to change answers on previous pages using a Back button.

Response rates

Unique site visitors: No IP addresses were stored and no cookies were used.

For every new first page visitor a unique code was generated. The total number of codes generated was 51,277. However, as mentioned above, the survey page was at certain times dysfunctional, which may have resulted in immediate discontinuation and later re-start of the survey. The first survey question was answered by 27,337 respondents; the last set of questions was answered by 14,392 respondents.

Due to the decision not to store IP addresses and not to use cookies, in combination with the technical difficulties during the implementation of the survey it is not possible to give meaningful numbers for the view rate and the participation rate. The completion rate can be calculated as 14,329/ 27,337 = 52%

Preventing multiple entries from the same individual

No technical tools such as cookies were used to prevent multiple entries from the same individual. However, due to the length of the questionnaire, technical capacity problems on the survey website which resulted in longer waiting times between screens further prolonging the time needed to fill in the questionnaire, and the lack of any material incentives, we think it is highly unlikely that individuals filled in the questionnaire more than once. It is however possible that respondents interrupted filling in the questionnaire and decided to restart at a later time point. To prevent using such possible multiple entries from the same individual, the final dataset was restricted to questionnaires in which at least the questions regarding gender, age, country, sexual orientation and HIV testing behaviour were answered. These represent the first approximately 10

page screens, and survey sections which did not include adaptive questions (27,337 respondents answered the first question on gender; 19,630 respondents answered the question on HIV testing).

IP check

No IP addresses of the client computer were used to identify potential duplicate entries from the same user.

Log file analysis

No other techniques to analyze the log file for identification of multiple entries were used.

Analysis

Incomplete questionnaires were also analysed when questions regarding gender, age, country, sexual orientation and HIV testing behaviour were answered.

Questionnaires submitted with an atypical timestamp

Time to fill out the questionnaire was not used as an exclusion criterion.

Statistical correction

No weighting of items or propensity scores have been used to adjust for the non-representative sample.

Annex I: Entry page of the online questionnaire

Subscriber information

Welcome to the survey!

Please take part in this survey if you ...

• are a gay man and / or

• are a man who feels attracted to men and / or

• are a man who has sex with men and

• are at least 16 years old.

We want to know it!

This survey, the study "Gay Men and HIV / AIDS 2013" refers to the sex you have, your knowledge and attitudes to HIV prevention and HIV testing and your life as a gay man, or a man who has sex with men, and how you are dealing with HIV and other sexually transmitted infections.

The questionnaire will take approximately 30 minutes.

Privacy Policy

Participation is anonymous. We guarantee that we will not save your IP address or collect information about you that could enable your identification by third parties.

For notes on the safe use of PCs, please refer to www.bsi.de.

Replying to the questionnaire is voluntary and can be cancelled at any time, without any disadvantages for you.

More information about objectives of this study can be found further down on this page.

Here we go!

Start the questionnaire by clicking on the following button:

[Button]

For more information on this study

We are psychologists and health scientists of the Free University Berlin. This study has been financed by the Federal Centre for Health Education (BZgA). For questions, comments or suggestions about the study please contact us at the e-mail address msm@zedat.fu-berlin.de

Goals

The primary objective of this survey is to obtain current information about how gay and other men who have sex with men (MSM) are dealing and living with HIV / AIDS and other sexually transmitted infections (STI). The collected answers will allow an assessment of the extent to which you and the

other participants protect themselves, but also what risks you are willing to take. Questions about the use of preventive services, and knowledge about progress in the treatability of HIV, will also allow to assess information needs and to better address these issues in HIV prevention. In addition to these points the general life situation of gay and other men who have sex with men living in Germany is an important part of this survey. In addition to dealing with discrimination against homosexuality, mental well-being is discussed in this survey for the first time. We want to investigate whether and why gay and bisexual men are more frequently affected by psychological stress. Also, the use of psychoactive substances (alcohol and drugs) will be investigated.

What happens to your data?

Taking into account the legal requirements of data protection we will evaluate your information together with that of the other participants to prepare scientific publications for a specialist audience. In this way we create the conditions that your information can be included in the optimization of prevention services for gay men and other men who have sex with men.

The central results of this survey can be expected to become available by autumn 2014, accessible on www.sma2013.de.

This study was reviewed by the ethics committee of the Charité Berlin, which confirmed the ethical acceptability of this study. The Data Protection Officer of the State of Berlin has examined the compliance with data protection and his suggestions for changes have been implemented.

Annex II: Invitation mail for men with a profile on planetromeo.com

Hello,

We would like to invite you to participate in a survey of gay and other men who have sex with men. This survey deals with your life, your sex and your relationships, your knowledge and attitudes to recent developments in HIV / AIDS, and how you are dealing with HIV and other sexually transmitted infections.

This survey is done anonymously and takes about 30 minutes. The survey is not optimized for filling in on smartphones. (Now start with the questionnaire!)

Your participation in this survey can not only help to ensure that you learn something new. Through your participation, you also support the prevention of HIV and other sexually transmitted diseases among gay and other men who have sex with men in Germany. The results of the study are directly feeding into this prevention work. In this way the prevention may take your needs better into consideration.

For more information about this study, please go to the home page of the questionnaire.

Your experiences and your vision are important to us. We would therefore be very happy if you participate in this survey: Click here for the questionnaire!

Scales and other measures

The questionnaire included two new ad-hoc scales describing HIV-related stigma of the respondents and anticipated stigma in case of being diagnosed with HIV and the validated internalized homonegativity scale.

1) HIV-stigma scale. Question: How would you deal with people who got infected with HIV?

This scale is an ad hoc scale which consists of six items:

(1) I wouldn't kiss somebody on the lips who is HIV positive
(2) I don' want to have direct contact with somebody who is HIV positive
(3) I can't imagine to have an HIV positive steady partner
(4) Someone who nowadays still gets infected with HIV must be stupid
(5) Somebody who nowadays still gets infected with HIV has deserved it
(6) HIV positive persons are irresponsible

[4 point disagree – agree scale]

For the calculation of the scale-value all items were coded, summed-up, and divided by the number of items. If any of the items was missing, the scale-value was set to missing. Scale values could be between 1 and 4, with higher scale values representing more stigmatising attitudes.

2) Anticipated Stigma. Question: How likely is it from your point of view that one of the following things will happen to you if you would become infected with HIV?

This scale is an ad hoc scale as well which consists of six items:

(1) Friends and acquaintances would think I failed
(2) Friends and acquaintances would blame me for getting infected
(3) My family would feel disappointed about me
(4) Potential sex partners would reject me
(5) Family members and friends would avoid me
(6) I would get problems with my job (with my education)

For the calculation of the scale-value all items were coded, summed-up, and divided by the number of items. If any of the items was missing, the scale-value was set to missing. Scale values could be between 1 and 4, with higher scale values representing more stigmatising attitudes.

[4 point likely – unlikely scale]

3) Internalized Homonegativity

Scale with 8 items, first item was left out. Maximum one missing item was allowed. The option “does not apply for me” was set to missing before calculating the scale.

(1) Obviously effeminate homosexual men make me feel uncomfortable.
(2) I feel comfortable in gay bars.
(3) Social situations with gay men make me feel uncomfortable.
(4) I feel comfortable being seen in public with an obviously gay person.
(5) I feel comfortable discussing homosexuality in a public situation.
(6) I feel comfortable being a homosexual man.
(7) Homosexuality is morally acceptable to me.
(8) Even if I could change my sexual orientation, I wouldn't.

[7 point disagree-agree with does not apply]

Higher scale values representing higher internalized homonegativity.

4) Subculture involvement

Gay subculture involvement was assessed by a question asking for the frequency of visiting venues such as (1) gay community centres, gay social groups, (2) cafes, bars, (3) discotheques or dance clubs (1-3 summarized as social venues), (4) bars with darkrooms, sex-clubs, public sex parties, (5) private sex parties, (6) gay saunas, porn cinemas, (7) other cruising sites such as parks or public toilets (4-7 summarized as sex venues), and (8) chat or dating websites or use of smartphone apps to meet new partners.

Frequency options of visiting these venues in the last 12 months options were several times per week, several times per month, several times per year, less frequent, and never. For analysis, frequencies of visits to physical venues (1-7) were dichotomized as frequent if visits were reported several times per month or more often, and the three other options were summarized as infrequent/never.

If any of the venues 1-3 was visited frequently, this was defined as ‘frequently visiting social venues’, if any of the venues 4-7 was visited frequently, this was defined as ‘frequently visiting sex venues’.
